# A Review on the Structure and Anti-Diabetic (Type 2) Functions of *β*-Glucans

**DOI:** 10.3390/foods11010057

**Published:** 2021-12-27

**Authors:** Yujun Wan, Xiaojuan Xu, Robert G. Gilbert, Mitchell A. Sullivan

**Affiliations:** 1 Centre for Nutrition and Food Sciences, Queensland Alliance for Agriculture and Food Innovation, The University of Queensland, Brisbane, QLD 4072, Australia; y.wan@uq.edu.au; 2 College of Chemistry and Molecular Sciences, Wuhan University, Wuhan 430072, China; xuxj@whu.edu.cn; 3 Joint International Research Laboratory of Agriculture and Agri-Product Safety, College of Agriculture, Yangzhou University, Yangzhou 225009, China; 4 Glycation and Diabetes Group, Mater Research Institute, The University of Queensland, Translational Research Institute, Brisbane, QLD 4072, Australia

**Keywords:** type 2 diabetes, *β*-glucan, molecular structure, anti-diabetic function, macronutrient absorption, enzyme inhibitor

## Abstract

Type 2 diabetes, a long-term chronic metabolic disease, causes severe and increasing economic and health problems globally. There is growing evidence that *β*-glucans can function as bioactive macromolecules that help control type 2 diabetes with minimal side effects. However, conflicting conclusions about the antidiabetic activities of *β*-glucans have been published, potentially resulting from incomplete understanding of their precise structural characteristics. This review aims to increase clarity on the structure–function relationships of *β*-glucans in treating type 2 diabetes by examining detailed structural and conformational features of naturally derived *β*-glucans, as well as both chemical and instrumental methods used in their characterization, and their underlying anti-diabetic mechanisms. This may help to uncover additional structure and function relationships and to expand applications of *β*-glucans.

## 1. Introduction

Type 2 diabetes (T2D), a disease associated with insulin resistance and poor blood glucose control, is a major public concern due to the potential severe complications and associated morbidity and its increasing rate of occurrence in many developed and developing countries. The major characteristic of this metabolic disease is chronic hyperglycemia, with severe complications leading to the long-term damage of various organs, including the eyes, kidneys, heart, blood vessels and nerves [1]. According to projections from the International Diabetes Federation, it has been estimated that in 2019 there were 463 million people with diabetes globally, and this is expected to reach 700 million by 2045, which will cost US$850 billion per year for diabetes healthcare [2,3]. Although several drugs have been used clinically to control T2D, such as biguanides [4], sulfonylureas [5], sodium-glucose co-transporter-2 (SGLT2) inhibitors [6], and thiazolidinediones [7], all of these drugs have some serious side-effects, especially resulting in gastrointestinal disorders [8,9] which affect both the drugs’ efficacy and the patient’s life. “Natural” medicine based on the concept of “food as medicine” has been proposed as an alternative strategy in the managing of metabolic diseases (such as T2D), due, among other things, to their safety [10]. Growing evidence has confirmed that certain bioactive nutrients in these foods, including polysaccharides [11,12], can help mitigate metabolic abnormalities [13].

Polysaccharides, one of the most important biomacromolecules for life, are polymers found in natural sustainable resources. From 1970, since the first discovery of the bioactive properties of lentinan, there has been a drive to research the biological functions of polysaccharides [14,15]. *β*-glucans are a type of naturally-derived polysaccharide which can be widely found in bacteria, alga, fungi, cereals, and higher plants. They are generally biopolymers with *β*-glucopyranosyl units, which normally contain a *β*-(1,3)-linked and/or *β*-(1,4)-linked backbone and may be branched with *β*-(1,6)-linked glucose. 

*β*-glucans have been used in the food industry and in clinical practice because of their significant biological functions. For example, *β*-glucans with high molecular weights have been reported to enhance the binding capacity to the receptors involved in immune responses (e.g., dectin-1: a natural killer (NK)-cell-receptor-like C-type lectin involving in innate immune responses) and therefore improve their immunomodulatory activities, which can help to control some chronic diseases such as diabetes [16]. However, many studies have reported that degrading *β*-glucans to yield lower molecular weights can increase their anti-diabetic effects *in vitro*, as well as their antioxidant and antibacterial activities [17,18]. These differences in reported results may arise from differences in the detailed molecular structures of the substrates [19]. Therefore, this paper reviews structural and conformational features of naturally derived *β*-glucans, summarizing the potential underlying mechanisms of their anti-diabetic functions, for a better understanding of the structure–function relationships of *β*-glucans.

## 2. Structural Features of *β*-Glucans

**Figure 1:**
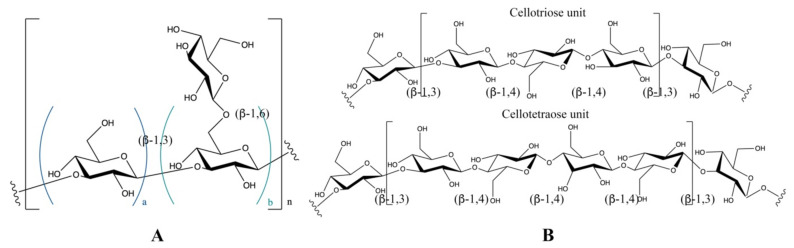
Chemical structures of *β*-glucans. The glucose monomers are shown following the symbol nomenclature for glycans. (**A**) The *β*-1,3-linked backbone of *β*-glucan with different branching degree of *β*-1,6-linked glucose. (**B**) The *β*-1,3-1,4-linked backbone of *β*-glucan, DP3: cellotriosyl, DP4: cellotetraosyl).

It is commonly asserted that the functionalities of *β*-glucans are highly dependent on their molecular structure. The structural characteristics of *β*-glucans, including molecular weight distributions, glycosidic linkage patterns and branching degrees, vary with different sources and extraction methods. There are three main glycosidic linkage patterns identified in *β*-glucans (Figure 1): *β*-1,3-linked, *β*-1,4-linked and *β*-1,6-linked patterns. Normally, *β*-1,3-linked and *β*-1,4-linked patterns appear in the backbone of *β*-glucan, and *β*-1,6- linkages represent the branch points in the backbone.

Branched or linear *β*-1,3-glucans (Figure 1A) are commonly isolated from fungi (e.g., mushroom [20]) and bacteria (e.g., yeast [20]). The occurrence of *β*-1,3-linkages together with *β*-1,4-linked glycosidic bonds (Figure 1B) are observed in the *β*-glucans from cereal grains (e.g., oat [21]). As shown in Figure 1B, there are two types of oligosaccharide subunits; one is three *β*-1,4-linked glucose monomers, termed cellotriosyl (DP3), and the other type is *β*-1,4-linked glucose monomers called cellotetraosyl (DP4). The molar ratio of DP3 and DP4 in *β*-glucans is specific for different cereals; this can be used as a tool to trace the origin of a given *β*-glucan structure [21].

The extraction methods of *β*-glucans vary from sample to sample. There are commonly four types of isolation method used in extracting *β*-glucans, such as water extraction [22,23,24], alkaline extraction [25,26], acidic extraction [27] and enzymatic extraction [28]. Some suggested structures of naturally derived *β*-glucans are shown in Table 1. These structures are generally inferred from a combination of results collected by both chemical tests and instrumental analysis. Interestingly, some similar repeating structural units exist in several *β*-glucans from different sources. For example, *β*-glucans from *Dictyophora indusiate*, *Hericium erinaceus*, *Grifola frondosa*, *Schizophyllan* and brown algae have the same repeating unit, viz., three *β*-1,3-Glc*p* backbone residues with a branch comprising one *β*-1,6-linked glucose residue. The branches of *β*-glucans, connected to the backbone via *β*-1,6 glycosidic bonds, play a major role in the solubility of the *β*-glucan. For example, curdlan, a linear *β*-glucan (i.e., without side chains) is insoluble in water [29], while the *β*-glucans with branched glucose residues, such as lentinan and Schizophyllan, are water-soluble [30,31]. However, these *β*-glucans exhibit some hydrophobicity due to hydrophobic carbon rings, resulting in limited water-solubility. Thus, *β*-glucans adopt different chain conformations to achieve stability. It is essential to consider their chain conformations in different solvents for the application of *β*-glucans in the food industry and medicine. 

## 3. Conformational Features of *β*-Glucans

**Figure 2:**
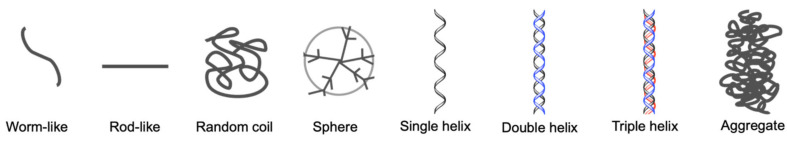
Various chain conformations of polysaccharides in different solvents.

Several chain conformations of *β*-glucans are found in different solutions (Figure 2), from a disordered conformation (e.g., random coil) to an ordered conformation (e.g., helix conformation). These more organized conformations can easily form a stable network (e.g., a triple helix), and the stabilization of this network arises from its inter- and intra-molecular hydrogen bonds. However, the dense triple helix conformation formed by the interaction of intramolecular polyhydroxy groups may result in its insolubility in aqueous solution [54].

There are many parameters that can affect conformational features of polysaccharides, including the molar mass per unit of contour length (*M*_L_), the contour length of chains (*L*), the persistence length (*q*) and the chain diameter (*d*). The contour length of a polymer chain refers to its length at maximum physically possible extension, and the persistence length reflects the bending stiffness of a chain. Several reports indicate that *β*-glucans that exhibit strong biological functions show a triple-helix conformation, such as lentinan [55], curdlan [56] and yeast *β*-glucan [57]. Based on both theoretical and experimental results, the *M*_L_ and *q* values of a polysaccharide with a rigid triple-helix conformation usually range from 2000 to 2800 nm^−1^ and from 100 to 250 nm, respectively [20]. For example, lentinan, the first reported *β*-glucan with antitumor activities, exists as a triple-helix conformation in aqueous solution, with a reported *M*_L_ value of 2160 nm^−1^ and *q* value of 110 nm [58]. Schizophyllan, a widely studied *β*-glucan from *Schizophyllum*, has a reported *M*_L_ value of 2150 nm^−1^ and *q* value of 200 nm [59]. However, the triple-helix conformation of these *β*-glucans can be transferred to other conformations under special conditions, such as high temperature [60], high pH solvents [61] and strong polar solvents [62]. With disrupted conformations, their bioactivities and solubilities are also changed. Therefore, the structural and conformational features of *β*-glucan need to be well characterized for a correct understanding of their functionalities.

## 4. Characterization Methods for *β*-Glucan Structure and Conformation Analysis

The structural determination of polysaccharides is more complicated than for other biopolymers due to the diverse monosaccharide compositions and glycosidic linkage patterns. In addition to classical chemical characterization methods, many newly developed technologies for the characterization of polysaccharides have been, or could be, employed to help understand the structure-function relationship of bioactive polysaccharides. For example, enzymatic arrays [63] and matrix-assisted laser desorption ionization mass spectrometry [64] have been used to sequence polysaccharides, and ion mobility-mass spectrometry has been developed to analyze carbohydrate anomers [65]. Advanced microscopy techniques, such as atomic force microscopy [66] and confocal laser scanning microscopy [67], provide a new level of microstructure analysis. Recently, low-temperature scanning tunneling microscopy has been successfully applied to observe single glycans [68]. However, the exploration of polysaccharides has been much slower than that of polynucleotides and proteins because of limitations on the structural theories, the complexity of their structures and a poor understanding of the underlying mechanisms of their bioactivities.

**Figure 3:**
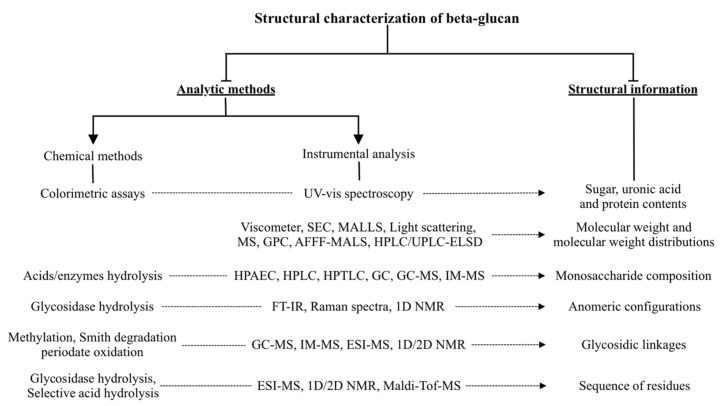
Chemical and instrumental methods used for *β*-glucan structure characterization.

The characterization of *β*-glucan structure therefore requires a combination of chemical and instrumental analyses (Figure 3). The structural information of *β*-glucans usually reported include its purity, molecular weight, monosaccharide composition, anomeric configuration, glycosidic linkage pattern and sequence of residues.

To investigate detailed structure–function relationships of polysaccharides, the “purity” is one of the most important factors. Generally, measuring the purity of *β*-glucans includes obtaining several parameters, such as the sugar content and the molecular weight distribution. Colorimetric methods are commonly used as the first step to determine the purity of crude polysaccharides, including the measurement of sugar, uronic acid and protein contents. Then, the molecular weight distributions of polysaccharides can be analyzed using several instrumental methods, such as size exclusion chromatography (SEC). Sometimes, *β*-glucans coexists with other polysaccharides, such as arabinogalactans [69]. Therefore, it is essential to analyze its monosaccharide composition to identify the purity of a *β*-glucan. Hydrolysis with acids or enzymes is the first step to analyze the monosaccharide composition, after which the hydrolysate is characterized with various instruments. High-performance anion exchange chromatography (HPAEC) is considered one of the most effective instrumental analysis techniques to determine monosaccharide composition due to the high sensitivity and simple sample preparation [70]. A high-purity *β*-glucan should have a narrow molecular weight distribution and high glucose content.

Extraction of *β*-glucans from grains always results in some starch (an *α*-glucan) [54], and it is difficult to distinguish *β*-glucans from *α*-glucans through their sugar content or monosaccharide composition alone. However, a combination of glycosidase hydrolysis and instrumental analysis, such as Raman spectra, FT-IR and NMR, can easily identify the anomeric configuration of glucans. The sequence of residue and the branching degree of naturally derived *β*-glucans is highly dependent on its source, and can be characterized using NMR. For a comprehensive characterization of naturally derived *β*-glucans, it is necessary to use both chemical methods and instrumental analysis.

To identify the conformation of *β*-glucans, weight-average molecular weight (*M_w_*), intrinsic viscosity ([*η*]), radius of gyration (*R*_g_) and hydrodynamic radius (*R*_h_) of *β*-glucan samples can be measured by static light scattering (SLS), dynamic light scattering (DLS) and viscometry, using the molecular-weight dependence of their solution properties. Several models, including the helical wormlike chains model and the Kratky-Porod model, can be used to deduce the four main parameters, *M*_L_, *L*, *q* and *d* based on the results of measurements.

Conformation analysis can be performed using X-ray diffraction (XRD), e.g., for measuring the triple-helix *β*-glucan [71]. Atomic force microscopy (AFM) is a powerful tool to observe chain conformations in solution, including rod-, sphere- and fiber-like shapes. AFM can provide information on the chain length, chain diameter and even the *M*_L_ of a *β*-glucan sample [58]. In addition to these experimental techniques, molecular dynamics simulations have been used as a tool to explore the conformation of polymers, which can help illustrate the chain movements and conformations of polymers in different solutions [32], although the results always depend on the assumed model. 

Although the characterization of *β*-glucans is complicated, an understanding of the structure/function relationships of these molecules is crucial if they are to advance further as a potential antidiabetic drug. Without this understanding the efficacy of a particular *β*-glucan is difficult to predict.

## 5. Amelioration of Type 2 Diabetes and Associated Mechanisms

T2D is the most frequent metabolic disorder which involves insulin resistance, followed by deficient insulin secretion by impaired pancreatic islet *β*-cells [72]. The two main factors that typically account for T2D are genetic factors and environmental factors. A genome-wide association study has confirmed that there are more than 400 gene variants associated with T2D, with most of them involving islet function [73]. The environmental factors that increase the risk of developing T2D include obesity, alcohol intake and smoking. The predominant factor accounting for T2D is the consumption of unhealthy diets, including those with energy-dense refined food [72].

### 5.1. Pharmacotherapy for T2D and Anti-Diabetic Mechanisms

There is a lack of effective drugs to treat T2D due to the complexity of pathogenesis [72,74]. However, several drugs are used in controlling T2D, and these drugs can be classified into seven main types based on their structures and mechanisms, including biguanides, sulfonylureas, thiazolidinediones, glucagon-like peptide (GLP-1), dipeptidyl peptidase (DPP-4) inhibitors, sodium-glucose co-transporter-2 (SGLT2) inhibitors and enzyme inhibitors. The underlying mechanisms and potential side-effects of these drugs are summarized in Table 2. As well as the injection of insulin being essential to control type 1 diabetes, this is also adopted to control T2D under certain conditions, such as functional failure of pancreatic islet *β*-cells due to the long-term suffering from T2D [75]. However, it should be noticed that all of these anti-diabetic drugs are companied by some severe side-effects (Table 2), such as gastrointestinal disorders, which have prolonged impact on the patient.

### 5.2. Glucans Used in Controlling T2D and Underlying Mechanisms

Naturally derived *β*-glucans have been promoted due to their various reported health-promoting activities and minimal side-effects. They have been widely adopted as health-improving ingredients to prevent some chronic diseases, especially for T2D. For example, a clinical trial showed that oat *β*-glucan can help manage glycemic index, carbohydrate metabolism and alter gut microbiota profile in T2D [89,90]. There are two main underlying mechanisms for the roles of *β*-glucans in controlling T2D, which can be explained through their detailed structural and/or conformational features. 

#### 5.2.1. Retardation of Macronutrient Absorption

Macronutrients within daily diets are necessary for life. However, ongoing quick absorption of these macronutrients in T2D can induce hyperglycemia and hyperlipidemia, and thereby cause metabolic disorders [91]. Hence, a way to help manage T2D is by preventing the absorption of macronutrients, resulting in a reduction in blood cholesterol levels and suppressing the postprandial increase of blood sugar levels [92]. This retardation effect of *β*-glucans has been shown to be highly dependent on their molecular weight and concentration. Wood et al. established the relationship of plasma glucose increment (D*G*pg) and structural features of oat *β*-glucan (concentration (*c*) and weight-average molecular weight (*M_w_*)) as shown in the formula: D*G*pg = *A* + *B* × log_10_(*c*) + 0.72B log_10_(*M_w_*) [93]. In addition, depolymerization of *β*-glucans (reducing molecular size) as a result of processing was reported to decrease its effect on decreasing the peak blood glucose response [17].

Additionally, *β*-glucans can play a role in increasing the viscosity of a meal during gastrointestinal digestion, limiting the absorption of macronutrients, slowing down gastric emptying, and entrapping bile acids and cholesterol throughout digestion. This lowers serum sugar and cholesterol levels in T2D [94]. These benefits are highly dependent on the structure and conformation of *β*-glucans, which can be explained by the Mark–Houwink equation for the intrinsic viscosity: [*η*] ∝ *K M*^α^, where the values of the parameters *K* and α depend on the particular polymer solution system [95].

#### 5.2.2. Inhibition of Digestive Enzyme

a-amylase and a-glucosidase are the two main enzymes necessary to hydrolyze carbohydrates in the digestive system. a-amylase can initiate carbohydrate hydrolysis by cleaving a-(1,4)-linked glycosidic bonds and yield smaller fractions, such as sucrose and maltose [96]. Then, a-glucosidase can hydrolyze these fractions into absorbable monosaccharides, such as glucose and fructose, during intestinal digestion [97]. Adequate free glucose can be generated after this digestive process, which may be excessively ingested into the bloodstream in T2D patients, leading to hyperglycemia. Therefore, inhibition of these enzymes to cause lower carbohydrate digestion can help control T2D. Ma et al. showed that a *β*-glucan adopting a triple helix conformation from *Hericium erinaceus* reduced wheat starch digestibility from 70% to less than 60% by inhibiting the digestive enzymes [98]. Qin et al. compared the glucose availability of the digestive system after adding different modified oat *β*-glucans, and all of these *β*-glucans decreased glucose availability, which indicated their potential hypoglycemic effect [17].

Lipases play an important role in hydrolyzing diet lipids; they convert lipids into cholesterol and fatty acids, which can be absorbed by enterocytes through lipid transporters [99]. Many studies have reported that *β*-glucans can inhibit lipase activity and therefore alleviate the hyperlipidemia seen in T2D. For example, barley *β*-glucans can slow lipolysis and reduce the release of free fatty acids; these inhibition effects were highly dependent on the molecular weights of the *β*-glucans [100].

**Figure 4:**
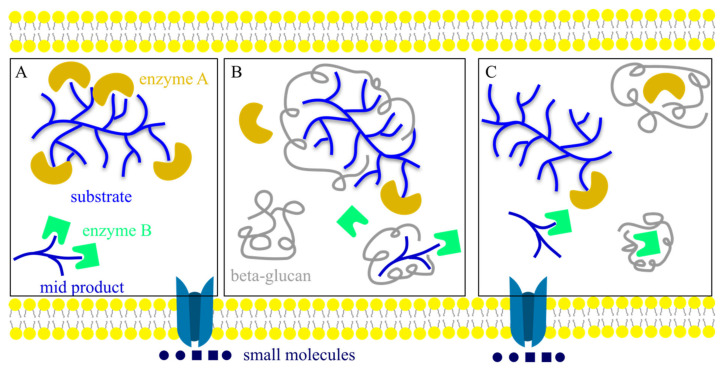
The proposed mechanism of enzyme inhibition. (**A**) The normal enzyme–substrate interactions during digestion. (**B**) *β*-glucans play as a physical barrier to inhibit enzyme–substrate interactions. (**C**) *β*-glucans bind to enzymes to inhibit enzyme–substrate interactions.

There are some possible underlying mechanisms related to the enzyme inhibitor role of *β*-glucans, as shown in Figure 4. For example, these *β*-glucans, which can only be fermented in the large intestine, play a physical barrier role in the digestive system to inhibit the activity of these digestive enzymes [101]. At the same time, *β*-glucans can inhibit these digestive enzymes by mixed competitive and uncompetitive inhibition to suppress enzyme–substrate interactions and therefore reduce the digestion of substrates [102]. Both of these proposed mechanisms rely on the physiological conformations of the *β*-glucans *in vivo*. For example, the binding between *β*-glucans and enzymes follows the lock-and-key principle; therefore, the shapes (confirmations) of *β*-glucans under physiological conditions are a decisive factor for binding efficiency, thereby controlling the inhibition effect of these *β*-glucans [20,103].

It should be noted that other possible anti-diabetic mechanisms of *β*-glucans have been proposed. For example, the modulation effects of *β*-glucan on gut microbiota have been widely reported [89,104]. *β*-glucans can function as prebiotics, as they are mainly fermented in the large bowel and benefit the host-microbiota interactions in the whole gastrointestinal process. As a prebiotic, *β*-glucans alter the microbiota compositions by improving the number of beneficial bacteria, such as *Lactobacillus*, during large bowel fermentation [105,106], leading to an increase in short-chain fatty acids (SCFA), which can improve the colonic defense barrier in T2D [107]. In addition, *β*-glucans can regulate the superoxide dismutase and malondialdehyde levels in the livers of diabetic mice [108]. However, these reported effects need to be further explored. For example some particular structural features of *β*-glucans may be preferred by these beneficial bacteria. Again a better understanding of the structure and function relationship of *β*-glucans may help yield more potent health benefits.

## 6. Conclusions

*β*-glucans are sustainable polymers that widely exist in natural resources. These biomacromolecules mainly contain *β*-(1,3)-linked, *β*-(1,4)-linked and *β*-(1,6)-linked glycosidic bonds, and adopt several different conformations in solutions, such as a helical conformation, which seems to be the origin of their versatile biofunctions and thus furnish a target for targeting efficacy. For a detailed characterization of *β*-glucans, both chemical and instrumental methods should be combined to give accurate structural and conformational features, which can then be linked to an understanding of their functionalities. Although there are many reported anti-diabetic mechanisms of *β*-glucans, only two mechanisms, retardation of macronutrient absorption and inhibition of digestive enzymes, can be well explained through their detailed structures and conformations. However, current research on the anti-diabetic functions of *β*-glucans is focused on naturally derived *β* -glucans. It would be worthwhile to explore the underlying anti-diabetic mechanisms using synthetic *β*-glucans with detailed structural information. With improved understanding of the structure/function relationship of these molecules, precision designs of *β*-glucans with particular structures and/or conformations could be produced to help control T2D.

## Figures and Tables

**Table 1:** Sources and deduced chemical structures of several *β*-glucans.

Name/Abbr.	Source	Extraction Solvent	Type of Glucan	Structure ^a^	Ref.
Curdlan	*Alcaligenes faecalis* var.	NaOH	*β*-1,3 glucan	(A) a = 1, b = 0	[25,26]
APP	*Auricularia auricula*	NaCl	β-1,3 glucan	(A) a = 1, b = 2	[32,33,34]
DIP	*Dictyophora indusiata*	Water	*β*-1,3 glucan	(A) a = 2, b = 1	[22,23,24]
HEP	*Hericium erinaceus*	Water	*β*-1,3 glucan	(A) a = 2, b = 1	[35,36]
GFP	*Grifola frondosa*	Water	*β*-1,3 glucan	(A) a = 2, b = 1	[37,38,39]
Schizophyllan	*Schizophyllum*	Water	*β*-1,3 glucan	(A) a = 2, b = 1	[30,40]
Laminarin	Algae	Water	*β*-1,3 glucan	(A) a = 2, b = 1	[41,42]
Lentinan	*Lentinula edodes*	NaCl/NaOH	*β*-1,3 glucan	(A) a = 3, b = 2	[31,43]
GLP	*Ganoderma lucidum*	Water	*β*-1,3 glucan	(A) a = 5, b = 1	[44,45]
YBG	*Saccharomyces cerevisiae*	NaOH	*β*-1,3 glucan	(A) a = 5, b = 1	[46]
CSP	Wild *Cordyceps sinensis*	Water	*β*-1,3 glucan	(A) a = 5, b = 2	[47]
WBG	Wheat	Water	*β*-1,3-1,4 glucan	(B) DP3:DP4 = 3.0–4.5	[21,48]
BBG	Barley	Water/NaOH	*β*-1,3-1,4 glucan	(B) DP3:DP4 = 1.7–3.3	[48,49]
RBG	Rye	Water	*β*-1,3-1,4 glucan	(B) DP3:DP4 = 1.8–3.1	[50,51,52]
OBG	Oat	Water	*β*-1,3-1,4 glucan	(B) DP3:DP4 = 1.5–2.2	[48,53]

^a^ The uppercase letters within this column represent the repeating units shown in Figure 1 (A. *β*-1,3-1,6 glucan; B. *β*-1,3-1,4 glucan). The lowercase letters indicate the molar ratio of each part in the repeating units.

**Table 2:** Drugs used in amelioration of T2D.

Type	Drug Name	Mechanisms	Side-Effects	Ref.
Biguanides	Metformin, Phenformin	Lowering fasting plasma insulin concentration; enhancing insulin sensitivity; changing gut microbiota composition; promoting functional shifts in gut microbiome.	Gastrointestinal disorders; folate deficiency; increasing homocysteine levels	[8,76,77]
Sulfonylureas	Glibenclamide, Glipizide	As insulin secretagogues to stimulate insulin secretion.	Gastrointestinal disorders, headache	[9,78,79]
Thiazolidinediones	Rosiglitazone, Pioglitazone	Improving insulin sensitivity by up-regulation of adipokine.	Peripheral and pulmonary edema; fluid retention.	[80,81]
GLP-1	Liraglutide, Semaglutide	Suppressing glucagon release; delaying gastric emptying and increasing satiety.	Nausea, vomiting and diarrhoea	[82,83]
DPP-4 inhibitors	Vidagliptin, Saxagliptin	Enhancing incretin axis; improving meal-stimulated insulin secretion by sparing incretin hormones.	Nausea and gastrointestinal problems	[78,84,85,86]
SGLT2 inhibitors	Dapagliflozin, Cangliflozin	Inhibition of renal glucose reabsorption to lower plasma glucose levers.	Increasing the risk of developing diabetic ketoacidosis.	[6,87]
Enzyme inhibitors	α-amylase inhibitors, α-glucosidase inhibitors,	Reduction in the rate of glucose absorption in post-prandial blood	Lactic acidosis, diarrhoea, liver function disorders.	[88]

## Data Availability

Not applicable.
